# Associação Positiva entre Autoanticorpos contra LDL Oxidada e HDL-C: Um Novo Mecanismo para Cardioproteção de HDL?

**DOI:** 10.36660/abc.20210796

**Published:** 2022-08-24

**Authors:** Carla Evelyn Coimbra Nunez, Joaquim Barreto Oliveira, Silvia de Barros-Mazon, Vanessa H. S. Zago, Denise Beheregaray Kaplan, Ruy T. Nakamura, Magnus Ake Gidlund, Erica I. L. Gomes, Patricia Miralda Cazita, Edna Nakandakare, Helison R. Carmo, Andrei C. Sposito, Eliana Cotta de Faria

**Affiliations:** 1 Universidade Estadual de Campinas Departamento de Patologia Campinas SP Brasil Universidade Estadual de Campinas (UNICAMP) – Departamento de Patologia , Campinas , SP – Brasil; 2 Universidade Estadual de Campinas Laboratório de Aterosclerose e Biologia Vascular Campinas SP Brasil Universidade Estadual de Campinas (UNICAMP) – Laboratório de Aterosclerose e Biologia Vascular (Atherolab), Campinas , SP – Brasil; 3 Pontifícia Universidade Católica Campinas SP Brasil Pontifícia Universidade Católica (PUC-Campinas), Campinas , SP – Brasil; 4 Diagnostic Image Laboratory Campinas SP Brasil Diagnostic Image Laboratory , Campinas , SP – Brasil; 5 Universidade de São Paulo São Paulo SP Brasil Universidade de São Paulo (USP), São Paulo , SP – Brasil; 6 Universidade Estadual de Campinas Campinas SP Brasil Universidade Estadual de Campinas (UNICAMP), Campinas , SP – Brasil

**Keywords:** HDL-Colesterol, Lipoproteínas IDL, Aterosclerose

## Abstract

**Fundamento:**

No microambiente da placa aterosclerótica, os fosfolipídios oxidados expressos na superfície de lipoproteína de baixa densidade oxidada (oxLDL) se ligam a receptores *scavenger* em macrófagos provocando a formação de células espumosas e a progressão da placa. Autoanticorpos contra oxLDL (oxLDL-Ab) interagem com epítopos oxidativos levando à formação de imunocomplexos que são incapazes de interagir com receptores de macrófagos, assim suprimindo a aterogênese. A liberação de oxLDL-Ab pelas células B envolve a resposta da interleucina 5 e Th2, que por sua vez são potencializadas pela HDL. Assim, levantamos a hipótese de que indivíduos com níveis mais altos de HDL-C podem apresentar níveis elevados de oxLDL-Ab.

**Objetivo:**

Avaliar a relação entre os níveis de HDL-C e oxLDL-Ab.

**Métodos:**

Indivíduos assintomáticos (n = 193) foram agrupados de acordo com sua concentração de HDL-C para uma das três categorias seguintes: baixa (< 68 mg/dL), intermediária (de 68 a 80 mg/dL) ou alta (> 80 mg/dL). Os valores p < 0,05 foram considerados estatisticamente significativos.

**Resultados:**

Nossa análise incluiu 193 indivíduos (média etária: 47 anos; masculino: 26,3%). Em comparação com os indivíduos no menor tercil de HDL-C, os mais elevados foram mais velhos (36 versus 53 anos; p = 0,001) e, menos frequentemente, masculinos (42,6% versus 20,9%; p = 0,001). Os valores médios de oxLDL-Ab aumentaram à medida que o grupo HDL-C aumentou (0,31, 0,33 e 0,43 unidades, respectivamente; p = 0,001 para tendência). A regressão linear simples encontrou uma relação significativa e positiva entre a variável independente, HDL-C, e a variável dependente, oxLDL-Ab (R = 0,293; p = 0,009). Essa relação manteve-se significativa (R = 0,30; p = 0,044), após ajuste por covariáveis. Os níveis de apolipoproteína AI também estiveram relacionados a oxLDL-Ab nos modelos de regressão linear simples e ajustada.

**Conclusões:**

HDL-C e oxLDL-Ab estão independentemente relacionados.

## Introdução

A acumulação de lipoproteínas contendo apolipoproteína B (ApoB), principalmente lipoproteína de baixa densidade (LDL), na íntima arterial tem sido pesquisada como o passo inicial da aterogênese.^1^ Nesse microambiente arterial, a modificação oxidativa gera vários novos epítopos na LDL, que são reconhecidas pelas células imunes e levam à ativação da resposta inflamatória Th1 e Th2, provocando a liberação de autoanticorpos contra LDL oxidada (oxLDL-Ab).^2 , 3^

O papel protetor de oxLDL-Ab na aterogênese é apoiado por um crescente corpo de evidências. De fato, o tratamento com oxLDL-Ab diminuiu a progressão da placa aterosclerótica e mitigou significativamente a captação da lipoproteína de baixa densidade oxidada (oxLDL) por macrófagos em camundongos com deficiência de apolipoproteína E (ApoE).^4^ Além disso, estudos observacionais descobriram que os níveis de oxLDL-Ab estão inversamente relacionados à espessura da camada íntima da carótida e aos níveis de oxLDL em indivíduos saudáveis.^8^ Da mesma maneira, uma grande revisão sistemática concluiu que os níveis de oxLDL-Ab estão inversamente relacionados à gravidade da doença arterial coronariana e à incidência de eventos cardiovasculares.^9^

As propriedades benéficas de oxLDL-Ab desencadearam uma intensa busca por moduladores de sua liberação. Neste assunto, Chou et al.^10^ verificaram que a estimulação de células B com interleucina 5 (IL5) induziu a geração de oxLDL-Ab. É importante ressaltar que a IL5 está relacionada à resposta Th2, que por sua vez é comprovadamente inibida por oxLDL, mas incrementada pela lipoproteína de alta densidade (HDL).^11 , 12^ Considerando isso, projetamos o presente estudo com a hipótese de que a HDL plasmática pode estar independentemente associada aos níveis plasmáticos de oxLDL-Ab. Portanto, nosso estudo avaliou se os níveis de oxLDL-Ab e colesterol de lipoproteína de alta densidade (HDL-C) estão relacionados em indivíduos com uma ampla variedade de concentrações plasmáticas de HDL-C.

## Métodos

### Desenho da pesquisa

O presente estudo foi realizado como análise transversal de dados de indivíduos saudáveis consecutivamente incluídos em um grande grupo de pacientes assintomáticos atendidos no Hospital Universitário da Universidade de Campinas, na cidade de Campinas, São Paulo, Brasil. Os pacientes elegíveis tinham 18 anos ou mais, sendo de ambos os sexos. Após a assinatura do termo de consentimento informado, os participantes responderam a um questionário detalhado de elegibilidade.

Os critérios de exclusão foram qualquer doença arterial coronariana prévia ou acidente vascular cerebral; causas secundárias de HDL-C plasmático baixo ou alto; uso regular de tratamentos médicos (especialmente aqueles que interferem no metabolismo lipídico, como estatinas, terapia de reposição hormonal e anticoncepcionais); doenças hepáticas, renais, pulmonares e endócrinas (como diabetes); uso crônico de álcool e tabaco; e mulheres que estavam grávidas ou lactantes devido à possível influência hormonal. Os participantes elegíveis foram submetidos a um exame físico detalhado, medidas de pressão arterial e ultrassonografia carotídea, e suas amostras de sangue periférico foram coletadas para análise bioquímica.

Os participantes foram agrupados de acordo com seus tercis de níveis de HDL-C da seguinte maneira: 1) baixas concentrações de HDL-C (HDL-C abaixo de 68 mg/dL: n = 59); 2) concentrações intermediárias (HDL-C 68 a 80 mg/dL: n = 71) e altas concentrações de HDL-C (HDL-C > 80 mg/dL: n = 63).

O Comitê de Ética em Pesquisa da Universidade Estadual de Campinas aprovou todos os procedimentos sob parecer número 790/2006. Todos os participantes assinaram uma declaração de consentimento para participar do estudo.

### Coleta de amostras e métodos analíticos

Foram coletadas amostras de sangue venoso após jejum de 12 horas nos indivíduos selecionados para participar do estudo. As amostras foram centrifugadas (4 °C, 1000 g, 10 minutos) para separação de soro e plasma EDTA e armazenadas a −80 °C até a análise. Foi coletada outra amostra de sangue em jejum de 12 horas durante uma segunda visita 15 minutos após a administração intravenosa de heparina (Liquemine® Roche, 100 U/kg de peso corporal).

Colesterol total, triglicerídeos e fosfolipídios no soro e os dois primeiros analitos em lipoproteínas foram analisados por métodos enzimático-colorimétricos (BM Hitachi 917 Roche, Mannheim, Alemanha). Foram medidas a apolipoproteína B100 e a apolipoproteína AI (ApoAI) em um sistema automatizado BN II (Siemens Healthcare Diagnostics, Marburg, Alemanha), utilizando ensaios comercialmente disponíveis (Dade-Boehringer®, Deerfield, Illinois, EUA). Foi analisado o HDL-C por um método direto homogêneo. O colesterol da lipoproteína de baixa densidade (LDL-C) foi calculado pela fórmula de Friedewald.^13^

Para obter as subfrações de HDL, as lipoproteínas que continham ApoB foram precipitadas por sulfato de dextrano, e o sobrenadante foi submetido a micro-ultracentrifugação sequencial utilizando o Airfuge/75B (Beckman Instruments, Palo Alto, California, EUA).

As atividades plasmáticas da proteína de transferência de éster de colesterol (CETP) e da proteína de transferência de fosfolipídios (PLTP) foram determinadas por meio de ensaios radiométricos usando substratos exógenos conforme descrito anteriormente.^14 , 15^ As atividades da lipase hepática (LH) e da lipoproteína lipase (LPL) foram medidas em amostras de plasma pós-heparina, coletadas 15 minutos após a administração intravenosa de heparina (100 U/kg de peso corporal). O ensaio foi baseado na liberação de ácidos graxos, utilizando uma emulsão de trioleína radiomarcada como substrato, e NaCl 1M como inibidor de LPL.^16^

Foi medida a proteína C-reativa de alta sensibilidade (hsCRP) por imunoturbidimetria utilizando o ensaio de alta sensibilidade Tina-quant® CRP (látex) (Roche Diagnostics®, Mannheim, Alemanha) na plataforma analítica Hitachi–Roche. Foi usado um kit ELISA comercial fabricado pela R&D para medir o fator de necrose tumoral alfa (TNF-α).

Foi usado o método ELISA para medir oxLDL-Ab no plasma de todos os participantes.^17 , 18^ Resumidamente, placas de microtitulação de poliestireno (Costar, Cambridge, Massachusetts, EUA) foram revestidas com 1 µg/ml de oxLDL humana (20 mM Cu^2^, 24 horas) em tampão carbonato/bicarbonato (20 µL/poço), pH 9,4, e armazenadas durante a noite a 4 ºC. As placas foram bloqueadas com uma solução de leite desnatado a 5% (Molico/Nestlé, São Paulo, Brasil) e, na sequência, incubadas por 2 horas em temperatura ambiente seguida por 4 lavagens com PBS (100 µL). Amostras de plasma (20 µL) foram adicionadas e as placas foram incubadas durante a noite a 4 ºC seguidas por lavagem com 1% Tween 20 em PBS. O anticorpo IgG anti-rato de coelho conjugado com peroxidase (20 µL; 1:1,500) foi então adicionado e, após 1 hora à temperatura ambiente, as placas foram lavadas. Em seguida, 75 µL de solução de substrato (250 mg de tetrametilbenzidina diluídos em 50 mL de DMSO, 10 µL de 30% H_2_O_2_, 12 mL de tampão citrato, pH 5,5) foram incorporados à mistura e, após incubação à temperatura ambiente por 15 minutos, a reação foi interrompida pela adição de 25 µL de ácido sulfúrico 2,0 M. A densidade óptica foi lida em um leitor de microplacas (Titertek Multiskan MCC/340P, modelo 2.20, Labsystems, Finlândia) a 450 nm.

Para todas as variáveis medidas, isto é, lipídios, marcadores inflamatórios e atividades enzimáticas, os coeficientes de variação intra/inter-ensaio aceitos variaram de 3% a 10% e de 10% a 15%, respectivamente.

### Análise estatística

Os dados são média ± desvio padrão para dados com distribuição normal e mediana (intervalo interquartil) para dados não paramétricos, enquanto as variáveis categóricas são apresentadas como número de casos (porcentagens). A normalidade das variáveis contínuas foi avaliada pelo teste de Kolmogorov-Smirnov. ANOVA de uma via com teste post hoc de Bonferroni e o teste Kruskal-Wallis com post hoc de Dunn-Bonferroni foram usados para comparar a distribuição de dados contínuos paramétricos e não paramétricos entre os grupos, respectivamente. O teste do qui-quadrado com ajuste de Bonferroni foi empregado para comparar a frequência entre os grupos de dados categóricos.

A regressão linear foi utilizada para avaliar a relação entre a variável independente, oxLDL-Ab, e a variável dependente, HDL-C. Este teste foi realizado após a verificação dos pressupostos de normalidade, linearidade, homocedasticidade e independência. A análise de regressão linear ajustada foi utilizada para avaliar a relação entre as variáveis independentes, HDL-C, ApoAI e HDL-3C, e a variável dependente, oxLDL-Ab, após ajuste por covariáveis. Os resultados apresentados como coeficientes de determinação (R^2^) representam a porcentagem de variação da variável dependente explicada pelas variáveis independentes. Valores de probabilidade (p) menores que 0,05 foram considerados estatisticamente significativos. Todas as análises foram realizadas no software SPSS versão 20.0 para Mac.

## Resultados

A Tabela 1 mostra as comparações das características clínicas, antropométricas e bioquímicas entre todos os subgrupos de HDL. Os participantes foram colocados, de acordo com suas concentrações de HDL-C, em uma das três categorias estatisticamente diferentes (p ≤ 0,006): baixa (< 60 mg/dL), intermediária (68 a 80 mg/dL) ou alta (> 80 mg/dL).

**Tabela 1:** Características clínicas, antropométricas e bioquímicas de indivíduos com concentrações diferentes de HDL-C

Parâmetros	Baixa (< 68 mg/dL)	Intermediária (68 a 80 mg/dL)	Alta (> 80 mg/dL)	Valor p
**n**	59	71	63	
**Idade (anos)**	36±14	52±12	53±13	*0,001* ^1,2^
**Sexo masculino (%)**	42,6	11,7	20,9	*0,001*
**IMC (kg/m^2^)**	24±5	25±5	25±5	*0,158*
**PAS (mmHg)**	119±13	124±14	130±18	*0,001* ^1,3^
**PAD (mmHg)**	77±9,2	79±10	83±11	*0,006* ^1^
**Colesterol (mg/dL)**	170±45	224±37	231±38	*0,001* ^1,2^
**TG (mg/dL)**	77 (38)	98 (39)	87 (40)	*0,008* ^2^
**Fosfolipídios (mg/dL)**	206±54	225±41	228±48	*0,023* ^1^
**HDL-C (mg/dL)**	51±5,6	73±3,9	86±4,7	*0,001* ^1,2,3^
**HDL_2_-C (mg/dL)**	11±2,9	17±4,6	19±4,7	*0,001* ^1,2,3^
**HDL_3_-C (mg/dL)**	36±6	55±7,6	64±6,7	*0,001* ^1,2,3^
**HDL_2_TG (mg/dL)**	6,8±4,8	8,6±4,7	24±9,8	*0,022* ^1^
**HDL_3_TG (mg/dL)**	51±5,5	73±4	86±4,7	*0,001* ^1,2^
**LDL-C (mg/dL)**	105±37	130±35	127±35	*0,001* ^1,2^
**VLDL (mg/dL)**	15±7,9	19±7,9	17±8,0	*0,020* ^2^
**ApoAI (mg/dL)**	140±27	180±24	195±31	*0,001* ^1,2,3^
**ApoB100 (mg/dL)**	82±30	104±25	101±26	*0,001* ^1,2^
**LH (nmol/AGL/mL/h)**	2529 (1361)	1539 (973)	1561 (1018)	*0,001* ^1,2^
**LPL (nmol/AGL/mL/h)**	2313 (1012)	2497 (1690)	2880 (1522)	*0,091*
**CETP (%)**	15,8±8,3	10,4±6,9	10,7±7,6	*0,001* ^1,2^
**PLTP (%)**	14,4±10,4	19,5±11,4	18,3±16,1	*0,020* ^2^
**hsCRP (mg/L)**	0,38±0,63	0,44±0,54	0,32±0,41	*0,451*
**TNF-α (pg/mL)**	9,8±9,3	11±12,3	12±12,1	*0,756*
**oxLDL-Ab (OD)**	0,31±0,17	0,33±0,16	0,43±0,17	*0,001* ^1^
**EMIC média (mm)**	0,62±0,12	0,90±0,24	0,86±0,22	*0,001* ^1,2^
**Uso de álcool % (n)**	16,1	24,7	28,4	*0,265*
**DAC % (n)**	9,4	6,8	9,0	0,866

*Os dados são representados como média ± desvio padrão e mediana (intervalo interquartil) quando distribuídos normalmente e não normalmente, respectivamente, e como número (%) quando categóricos. Baixa: HDL-C < 68; intermediária: HDL-C 68 a 80 mg/dL; alta: HDL-C > 80 mg/dL. AGL: ácidos graxos livres; ApoAI: apolipoproteína AI; ApoB100: apolipoproteína B100; C: colesterol; CETP: proteína de transferência de éster de colesterol; DAC: doença arterial coronariana; EMIC: espessura médio-intimal carotídea; HDL: lipoproteína de alta densidade; hsCRP: proteína C-reativa de alta sensibilidade; IMC: índice de massa corporal; LDL: lipoproteína de baixa densidade; LH: lipase hepática; LPL: lipoproteína lipase; PAD: pressão arterial diastólica; PAS: pressão arterial sistólica; oxLDL-Ab: autoanticorpos contra lipoproteína de baixa densidade oxidada; PLTP: proteína de transferência de fosfolipídios; TG: triglicerídeos; TNF-α: fator de necrose tumoral alfa; VLDL: lipoproteína de muito baixa densidade. Os valores de p foram obtidos por ANOVA de uma via e teste de Kruskal-Wallis para variáveis contínuas com distribuição normal e não normal, e pelo teste qui-quadrado para dados categóricos. As diferenças entre os grupos são representadas por ^1^(baixo ≠ alto), ^2^(baixo ≠ intermediário), ^3^(intermediário ≠ alto).*

Quando comparado ao tercil mais baixo de HDL-C, o tercil mais alto apresentava mais mulheres, idades mais avançadas e, como esperado, maior concentração de colesterol. As atividades de LH e CETP foram reduzidas, e LH e PLTP aumentaram no tercil mais alto de HDL-C em comparação com o tercil mais baixo. Não foram encontradas diferenças em hsCRP e TNFα. Vale ressaltar que os níveis de oxLDL-Ab foram significativamente maiores entre no grupo com HDL-C alta, em comparação ao grupo com HDL-C baixa.

Para explorar a influência das variáveis independentes, concentração de HDL-C, tercis de HDL-C, sexo, ApoAI, ApoB, marcadores inflamatórios e idade, na variável dependente, oxLDL-Ab, foi realizada uma análise de regressão linear, conforme mostrado na Tabela 2 . Os níveis de OxLDL-Ab foram influenciados pela idade, HDL-C, tercis de HDL-C, HDL-3C e ApoAI.

**Tabela 2:** Regressão linear simples usando oxLDL-Ab como variável dependente

Variáveis independentes	B (SE)	Valor p	R	R^2^
**Idade**	0,002 (,001)	**0,027**	0,216	0,047
**Sexo masculino**	8,66 (,029)	0,998	0,000	0,000
**HDL-C**	0,002 (,001)	**0,004**	0,293	0,086
**Tercis de HDL**	0,042 (,015)	**0,006**	0,276	0,076
**HDL-2C**	0,004 (,002)	0,054	0,191	0,036
**HDL-3C**	0,002 (,001)	**0,016**	0,237	0,056
**ApoAI**	0,001 (,000)	**0,002**	0,308	0,095
**LDL**	0,000 (,000)	0,179	0,132	0,017
**ApoB**	0,001 (,000)	0,204	0,126	0,016
**hsCRP**	0,052 (,032)	0,105	0,177	0,031
**TNF-α**	0,002 (,001)	0,086	0,225	0,051
**EMIC**	0,146 (,085)	0,093	-0,25	0,316

*Regressão linear simples. ApoAI: apolipoproteína AI; ApoB: apolipoproteína B; C: colesterol; EMIC: espessura médio-intimal da carótida; HDL: lipoproteína de alta densidade; hsCRP: proteína C reativa de alta sensibilidade; LDL: lipoproteína de baixa densidade; oxLDL-Ab: autoanticorpos contra lipoproteína de baixa densidade oxidada; TNF-α: fator de necrose tumoral alfa.*

Na análise de regressão ajustada, apenas HDL-C e ApoAI foram independentemente relacionados aos níveis de oxLDL-Ab no modelo ajustado pelas covariáveis de idade e ApoB, conforme mostrado na Tabela 3 .

**Tabela 3:** Regressão linear ajustada por covariáveis

Variáveis independentes	Modelos	B (SE)	Valor p	R	R^2^
**HDL-C**	**HDL-C** Idade	0,002 (,001)	0,044	0,30	0,090
	**HDL-C** ApoB	0,002 (,001)	0,011	0,279	0,078
**HDL-3C**	**HDL-3C** Idade	0,001 (,001)	0,166	0,272	0,074
	**HDL-3C** ApoB	**0,002 (,001)**	0,054	**0,234**	**0,055**
**ApoAI**	**ApoAI** Idade	0,153 (,059)	0,019	0,318	0,101
	**ApoAI** ApoB	0,001 (,00)	0,004	0,310	0,096

*Regressão linear ajustada. ApoAI: apolipoproteína AI; ApoB: apolipoproteína B; HDL-C: colesterol de lipoproteína de alta densidade.*

**Figura 1:**
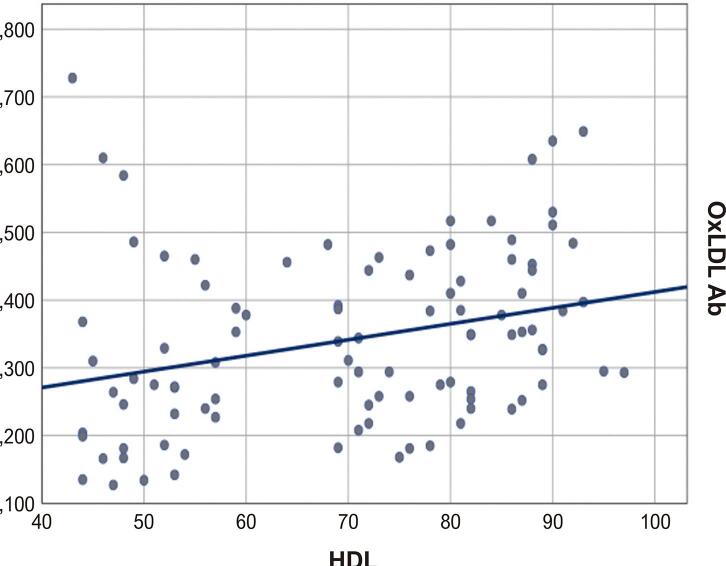
Modelo de curva: oxLDL-Ab versus HDL. HDL: lipoproteína de alta densidade; oxLDL-Ab: autoanticorpos contra lipoproteína de baixa densidade.

Modelos de curva de regressão linear das relações de HDL e ApoAI com oxLDL-Ab são mostrados nas Figuras 1 e 2 , respectivamente.

**Figura 2:**
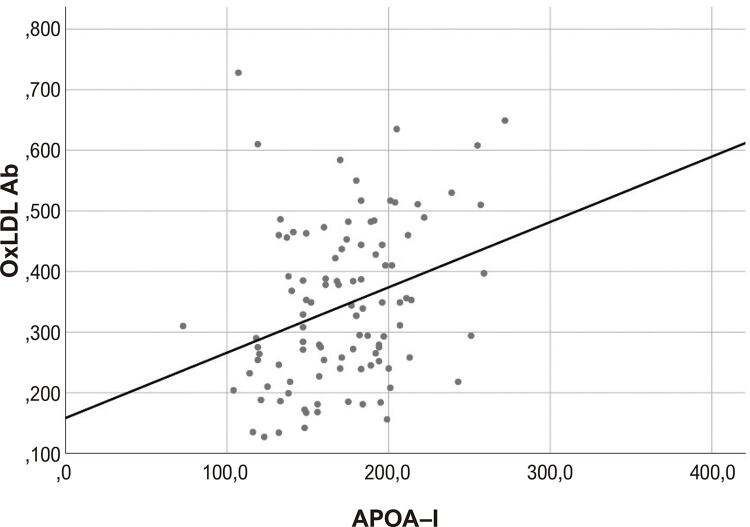
Modelo de curva: oxLDL-Ab versus ApoAI. ApoAI: apolipoproteína AI; oxLDL-Ab: autoanticorpos contra lipoproteína de baixa densidade.

## Discussão

A retenção de oxLDL na camada subendotelial da parede arterial é um passo inicial da aterosclerose.^19^ A oxLDL liga-se a receptores *scavenger* , como Lox1 e SR-B1, para desencadear vias deletérias que culminam na progressão da placa.^20^ Além do transporte reverso de colesterol, a HDL modula a imunidade humoral da placa aterosclerótica, regulando positivamente a resposta de IL5 e Th2, que estão envolvidas na ativação de células B e na liberação de oxLDL-Ab.^21^ Correspondentemente, nosso estudo, pela primeira vez, encontrou uma correlação positiva independente entre níveis séricos de HDL-C e oxLDL-Ab.

Dados experimentais anteriores demonstraram consistentemente um papel ateroprotetor para oxLDL-Ab. De uma perspectiva mecanicista, oxLDL-Ab coloca-se na placa aterosclerótica, onde se liga a epítopos de oxLDL, formando complexos imunes que não podem interagir com os receptores Fcγ de macrófagos.^13 , 22^ Como resultado, a neutralização de epítopos de oxLDL por oxLDL-Ab impede a ativação de macrófagos, interrompendo uma via imperativa de progressão da placa.^13^ Em consonância com isso, Dai et al.^23^ demonstraram que o pré-tratamento de macrófagos com oxLDL-Ab preveniu a morte celular induzida por oxLDL e a ativação de NF-kappaB. De acordo com isso, o tratamento com oxLDL-Ab significativamente reduziu a área transversal da placa aterosclerótica e a molécula de adesão celular vascular 1, e mitigou a captação de macrófagos em camundongos LDLr^-^

Dados cumulativos de estudos clínicos também apoiaram o papel de oxLDL-Ab como marcador de doença cardiovascular. A esse respeito, os níveis séricos de oxLDL-Ab consistentemente mostraram uma correlação inversa independente com a espessura íntima-média da artéria carótida comum e a progressão da aterosclerose carotídea.^16 , 25 - 30^ Por exemplo, em uma coorte de 226 pacientes com hipertensão inscritos prospectivamente na análise por ultrassonografia carotídea, aqueles com menor valor de oxLDL-Ab mostraram um risco 3 vezes menor que qualquer progressão da espessura íntima-média das artérias carótidas ao longo 4 anos.^31^ De forma semelhante, entre os indivíduos submetidos à angiografia coronária clinicamente indicada, aqueles nos tercis mais altos de oxLDL-Ab tiveram um risco 37% menor de aterosclerose coronariana angiograficamente significativa e apresentaram um número menor de artérias com doença quando comparados àqueles com os níveis mais baixos de anticorpos.^32 , 33^ Consistentemente, Shoji et al.^34^ observaram um aumento de 2 vezes na mortalidade cardiovascular em 5 anos entre indivíduos com doença renal em estágio final e baixo oxLDL-Ab, quando comparados a pacientes com doença renal em estágio final com níveis mais elevados de oxLDL-Ab.

Além do exposto, verificamos uma correlação independente positiva entre os níveis séricos de HDL-C e oxLDL-Ab. De uma perspectiva mecanicista, esse achado pode derivar dos efeitos imunomoduladores de HDL na resposta Th2, que razoavelmente potencializa a liberação de oxLDL-Ab. Essa hipótese ainda merece uma investigação mais profunda. Outras razões potenciais para a correlação verificada podem ser destacadas, por exemplo, experimentalmente, a captação de HDL atenuada de oxLDL por macrófagos. Isso pode resultar no acúmulo de oxLDL no microambiente da placa, favorecendo a resposta humoral local.^35^

O presente estudo teve algumas limitações. Mais importante, assumimos que HDL induz oxLDL-Ab modulando a resposta Th2 relacionada a IL5. No entanto, não foi realizada a medição da IL5. Além disso, os níveis de oxLDL, que estão intimamente relacionados à liberação de oxLDL-Ab, também não foram avaliados e teriam sido uma variável de ajuste razoável em nossos modelos. Por fim, o tamanho da amostra foi relativamente pequeno, o que pode ter comprometido o poder estatístico para afirmar a correlação.

## Conclusão

Os níveis séricos de oxLDL-Ab e HDL-C estão positivamente relacionados.
