# Supplementary material for: Associação Positiva entre Autoanticorpos contra LDL Oxidada e HDL-C: Um Novo Mecanismo para Cardioproteção de HDL?
Source: Arq Bras Cardiol. 2022 Aug 24;119(5):714–21. [Article in Portuguese] doi: 10.36660/abc.20210796 (PMC9750204; doi:10.36660/abc.20210796)
Supplement: Translation

# Translation: Positive Association between Autoantibodies Against Oxidized LDL and HDL-C: A Novel Mechanism for HDL Cardioprotection?

**Authors:** Carla Evelyn Coimbra Nunez, Joaquim Barreto Oliveira, Silvia de Barros-Mazon, Vanessa H. S. Zago, Denise Beheregaray Kaplan, Ruy T. Nakamura, Magnus Ake Gidlund, Erica I. L. Gomes, Patricia Miralda Cazita, Edna Nakandakare, Helison R. Carmo, Andrei C. Sposito, Eliana Cotta de Faria

**Affiliations:** 1 Brazil Universidade Estadual de Campinas (UNICAMP) – Departamento de Patologia, Campinas, SP – Brazil; 2 Brazil Universidade Estadual de Campinas (UNICAMP) – Laboratório de Aterosclerose e Biologia Vascular (Atherolab), Campinas, SP – Brazil; 3 Brazil Pontifícia Universidade Católica (PUC-Campinas), Campinas, SP – Brazil; 4 Brazil Diagnostic Image Laboratory, Campinas, SP – Brazil; 5 Brazil Universidade de São Paulo (USP), São Paulo, SP – Brazil; 6 Brazil Universidade Estadual de Campinas (UNICAMP), Campinas, SP – Brazil

**Keywords:** Cholesterol, HDL, Lipoproteins, IDL, Atherosclerosis

## Abstract

**Background:**

In the atherosclerotic plaque microenvironment, oxidized phospholipids expressed in the oxidized low-density lipoprotein (oxLDL) surface bind to scavenger receptors of macrophages eliciting foam cell formation and plaque progression. Auto-antibodies against oxLDL (oxLDL-Ab) interact with oxidative epitopes leading to the formation of immune complexes that are unable to interact with macrophage receptors, thus abrogating atherogenesis. Release of oxLDL-Ab by B cells involves interleukin 5 and Th2 response, which in turn are potentiated by HDL. Thereby, we hypothesized that individuals with higher levels of HDL-C may plausibly display elevated titers of oxLDL-Ab.

**Objective:**

To evaluate the relationship between HDL-C and oxLDL-Ab levels.

**Methods:**

Asymptomatic individuals (n = 193) were grouped according to their HDL-C concentration to one of three categories: low (< 68 mg/dL), intermediate (68 to 80 mg/dL) or high (> 80 mg/dL). P values < 0.05 were considered statistically significant.

**Results:**

Our analysis included 193 individuals (mean age: 47 years; male: 26.3%). Compared to individuals in the lowest HDL-C tertile, those in the highest tertile were older (36 versus 53 years; p = 0.001) and less frequently male (42.6% versus 20.9%; p = 0.001). Mean values of oxLDL-Ab increased as the HDL-C group escalated (0.31, 0.33 and 0.43 units, respectively; p = 0.001 for trend). Simple linear regression found a significant, positive relationship between the independent variable, HDL-C, and the dependent variable, oxLDL-Ab (R = 0.293; p = 0.009). This relation remained significant (R = 0.30; p = 0.044), after adjustment by covariates. Apolipoprotein AI levels were also related to oxLDL-Ab in both simple and adjusted linear regression models.

**Conclusion:**

HDL-C and oxLDL-Ab are independently related.

## Introduction

Accumulation of apolipoprotein B (ApoB)-containing lipoproteins, chiefly low density lipoprotein (LDL), in arterial intima has been pursued as the initial step of atherogenesis.^1^ In this arterial microenvironment, oxidative modification generates several new epitopes in LDL, which are recognized by immune cells and lead to the activation of Th1 and Th2 inflammatory response eliciting the release of autoantibodies against oxidized LDL (oxLDL-Ab).^2 , 3^

The protective role of oxLDL-Ab in atherogenesis is supported by a growing body of evidence. In fact, treatment with oxLDL-Ab diminished atherosclerotic plaque progression, and significantly mitigated oxidized LDL (oxLDL) uptake by macrophages in apolipoprotein E (ApoE)-deficient mice.^4 - 7^ Moreover, observational studies have found that oxLDL-Ab levels are inversely related to carotid intima media thickness and to oxLDL levels in healthy individuals.^8^ Accordingly, a large systematic review concluded that oxLDL-Ab levels are inversely related to coronary artery disease severity and incidence of cardiovascular events.^9^

The beneficial properties of oxLDL-Ab have sparked an intense search for modulators of its release. In this matter, Chou et al.^10^ found that stimulation of B cells with interleukin 5 (IL5) elicited generation of oxLDL-Ab. Importantly, IL5 is related to Th2 response, which in turn is proven to be inhibited by oxLDL but enhanced by high-density lipoprotein (HDL).^11 , 12^ This considered, we designed the present study hypothesizing that plasma HDL may be independently associated with plasma levels of oxLDL-Ab. To address this, our study evaluated whether oxLDL-Ab and high-density lipoprotein cholesterol (HDL-C) levels are related in individuals with a large range of plasma HDL-C concentrations.

## Methods

### Research design

The study was conducted as cross-sectional analysis of data from healthy individuals consecutively enrolled in a large pool of asymptomatic patients attended at the University of Campinas Teaching Hospital, in the city of Campinas, Sao Paulo, Brazil. Eligible patients were 18 years or older, from both sexes. After signing the informed consent form, participants answered a detailed eligibility questionnaire.

Exclusion criteria were any previous coronary artery disease or stroke; secondary causes of low or high plasma HDL-C; regular use of medical treatments (especially those interfering in lipid metabolism, such as statins, hormonal replacement therapy, and contraceptives); liver, renal, lung, and endocrine diseases (such as diabetes); chronic use of alcohol and tobacco; and women who were pregnant or lactating due to the possible hormone influence. Eligible participants then underwent a detailed physical examination, blood pressure measurements, and carotid ultrasound examination, and their peripheral blood sample was collected for biochemical analysis.

Participants were grouped according to their HDL-C levels tertiles as follows: 1) low HDL-C concentrations (HDL-C below 68 mg/dL: n = 59); 2) intermediate concentrations (HDL-C 68 to 80 mg/dL: n = 71) and high HDL-C concentrations (HDL-C > 80 mg/dL: n = 63).

The Research Ethics Committee of the State University of Campinas approved all procedures under opinion number 790/2006. All participants signed a consent declaration to participate in the study.

### Sample collection and analytical methods

Venous blood samples were drawn after a 12-hour fasting period in individuals selected to participate in the study. The samples were centrifuged (4 °C, 1000 g, 10 minutes) for serum and EDTA plasma separation, and stored at −80 °C until analysis. Another 12-hour fasting blood sample was collected during a second visit 15 minutes after intravenous administration of heparin (Liquemine® Roche, 100 U/kg body weight).

Total cholesterol, triglycerides, and phospholipids in serum and the first two analytes in lipoproteins were analyzed by enzymatic-colorimetric methods (BM Hitachi 917 Roche, Mannheim, Germany). Apolipoprotein B100 and apolipoprotein AI (ApoAI) were measured in an automated BN II system (Siemens Healthcare Diagnostics, Marburg, Germany), using commercially available assays (Dade-Boehringer®, Deerfield, Illinois, USA). HDL-C was analyzed by a direct homogenous method. Low-density lipoprotein cholesterol (LDL-C) was calculated by Friedewald’s^13^ formula.

To obtain HDL sub-fractions, lipoproteins that contained ApoB were precipitated by dextran sulfate, and the supernatant substance was submitted to sequential micro-ultracentrifugation using the Airfuge/75B (Beckman Instruments, Palo Alto, California, USA).

Plasma activities of cholesteryl ester transfer protein (CETP) and phospholipid transfer protein (PLTP) were determined through radiometric assays using exogenous subtracts as previously described.^14 , 15^ Hepatic lipase (HL) and lipoprotein lipase (LPL) activities were measured in post-heparin plasma samples, collected 15 minutes after intravenous administration of heparin (100 U/kg of body weight). The assay was based on the fatty acid release, using a radiolabeled triolein emulsion as substrate, and NaCl 1M as LPL inhibitor.^16^

High-sensitivity C-reactive protein (hsCRP) was measured by immunoturbidimetry utilizing the Tina-quant® CRP (latex) high sensitivity assay (Roche Diagnostics®, Mannheim, Germany) in the Hitachi–Roche analytical platform. A commercial ELISA kit manufactured by R&D was used for tumor necrosis factor alpha (TNF-α) measurement.

The ELISA method was used to measure oxLDL-Ab in the plasma of all participants.^17 , 18^ Briefly, polystyrene microtiter plates (Costar, Cambridge, Massachusetts, USA) were coated with 1 µg/ml of human oxLDL (20 mM Cu^2^, 24 hours) in carbonate/bicarbonate buffer (20 µL/well), pH 9.4, and kept overnight at 4 ºC. The plates were blocked with a 5% solution of fat-free milk (Molico/Nestlé, São Paulo, Brazil), and then incubated for 2 hours at room temperature followed by washing 4 times with PBS (100 µL). Plasma samples (20 µL) were added, and the plates were incubated overnight at 4 ºC followed by washing with 1% Tween 20 in PBS. The peroxidase-conjugated rabbit anti-mouse IgG antibody (20 µL; 1:1.500) was then added, and after 1 hour at room temperature, the plates were washed. Subsequently, 75 µL of substrate solution (250 mg of tetramethylbenzidine diluted in 50 mL of DMSO, 10 µL of 30% H_2_O_2_, 12 mL of citrate buffer, pH 5.5) were incorporated to the mixture and, after incubation at room temperature for 15 minutes, the reaction was stopped by adding 25 µL of 2.0 M sulfuric acid. The optical density was read in a microplate reader (Titertek Multiskan MCC/340P, model 2.20, Labsystems, Finland) at 450 nm.

For all measured variables, i.e., lipid, inflammatory markers, and enzyme activities, the accepted intra/inter-assay coefficients of variation varied from 3% to 10%, and from 10% to 15%, respectively.

### Statistical analysis

Data are mean ± standard deviation for normally distributed data and median (interquartile range) for non-parametric data, whereas categorical variables are presented as number of cases (percentages). The normality of the continuous variables was assessed by the Kolmogorov-Smirnov test. One-way ANOVA with Bonferroni post-hoc test and Kruskal-Wallis with Dunn-Bonferroni post hoc were used to compare the distribution of parametric and nonparametric continuous data across groups, respectively. Chi-square test with Bonferroni adjustment was employed to compare the frequency across groups of categorical data.

Linear regression was utilized to assess the relationship between the independent variable, oxLDL-Ab, and the dependent variable, HDL-C. This test was performed after assumptions of normality, linearity, homoscedasticity, and independence were ascertained. Adjusted linear regression analysis was utilized to assess the relationship between the independent variables, HDL-C, ApoAI, and HDL-3C, and the dependent variable, oxLDL-Ab, after adjustment by covariates. The results shown as coefficients of determination (R^2^) represent the percentage of variation in the dependent variable explained by the independent variables. Probability values (p) less than 0.05 were considered statistically significant. All analyses were performed using the software SPSS version 20.0 for Mac.

## Results

Table 1 shows the comparisons of clinical, anthropometric, and biochemical characteristics among all HDL subgroups. The participants were assigned, according to their HDL-C concentrations to one of three statistically different categories (p ≤ 0.006): low (< 60 mg/dL), intermediate (68 to 80 mg/dL) or high (> 80 mg/dL).

**Table 1:** Clinical, anthropometric, and biochemical characteristics of individuals with different concentrations of HDL-C

Parameters	Low (< 68 mg/dL)	Intermediate (68 to 80 mg/dL)	High (> 80 mg/dL)	p values
**n**	59	71	63	
**Age (years)**	36±14	52±12	53±13	*0.001* ^1,2^
**Male (%)**	42.6	11.7	20.9	*0.001*
**BMI (kg/m^2^)**	24±5	25±5	25±5	*0.158*
**SBP (mmHg)**	119±13	124±14	130±18	*0.001* ^1,3^
**DBP (mmHg)**	77±9.2	79±10	83±11	*0.006* ^1^
**Cholesterol (mg/dL)**	170±45	224±37	231±38	*0.001* ^1,2^
**TG (mg/dL)**	77 (38)	98 (39)	87 (40)	*0.008* ^2^
**Phospholipids (mg/dL)**	206±54	225±41	228±48	*0.023* ^1^
**HDL-C (mg/dL)**	51±5.6	73±3.9	86±4.7	*0.001* ^1,2,3^
**HDL2-C (mg/dL)**	11±2.9	17±4.6	19±4.7	*0.001* ^1,2,3^
**HDL3-C (mg/dL)**	36±6	55±7.6	64±6.7	*0.001* ^1,2,3^
**HDL2TG (mg/dL)**	6.8±4.8	8.6±4.7	24±9.8	*0.022* ^1^
**HDL3TG (mg/dL)**	51±5.5	73±4	86±4.7	*0.001* ^1,2^
**LDL-C (mg/dL)**	105±37	130±35	127±35	*0.001* ^1,2^
**VLDL (mg/dL)**	15±7.9	19±7.9	17±8.0	*0.020* ^2^
**ApoAI (mg/dL)**	140±27	180±24	195±31	*0.001* ^1,2,3^
**ApoB100 (mg/dL)**	82±30	104±25	101±26	*0.001* ^1,2^
**HL (nmol/FFA/mL/h)**	2529 (1361)	1539 (973)	1561 (1018)	*0.001* ^1,2^
**LPL (nmol/FFA/mL/h)**	2313 (1012)	2497 (1690)	2880 (1522)	*0.091*
**CETP (%)**	15.8±8.3	10.4±6.9	10.7±7.6	*0.001* ^1,2^
**PLTP (%)**	14.4±10.4	19.5±11.4	18.3±16.1	*0.020* ^2^
**hsCRP (mg/L)**	0.38±0.63	0.44±0.54	0.32±0.41	*0.451*
**TNF-α (pg/mL)**	9.8±9.3	11±12.3	12±12.1	*0.756*
**oxLDL-Ab (OD)**	0.31±0.17	0.33±0.16	0.43±0.17	*0.001* ^1^
**Mean CIMT (mm)**	0.62±0.12	0.90±0.24	0.86±0.22	*0.001* ^1,2^
**Alcohol use % (n)**	16.1	24.7	28.4	*0.265*
**CAD % (n)**	9.4	6.8	9.0	0.866

Data are represented as mean ± standard deviation and median (interquartile range) when normally and non-normally distributed, respectively, and as number (%) when categorical. Low: HDL-C < 68; intermediate: HDL-C 68 to 80 mg/dL; high: HDL-C > 80 mg/dL. ApoAI: apolipoprotein AI; ApoB100: apolipoprotein B100; BMI: body mass index; C: cholesterol; CAD: coronary artery disease; CETP: cholesterol ester transfer protein; CIMT: carotid intima-media thickness; DBP: diastolic blood pressure; FFA: free fatty acids; HDL: high-density lipoprotein; HL: hepatic lipase; hsCRP: high-sensitivity C-reactive protein; LDL: low-density lipoprotein; LPL: lipoprotein lipase; oxLDL-Ab: autoantibodies against oxidized low-density lipoprotein; PLTP: phospholipid transfer protein; SBP: systolic blood pressure; TG: triglycerides; TNF-α: tumor necrosis factor alpha; VLDL: very low-density lipoprotein. The p values were obtained by one-way ANOVA and Kruskal-Wallis test for continuous variables with normal and non-normal distribution, and by chi-square test for categorical data. Differences among groups are represented by^1^(low ≠ high),^2^(low ≠ intermediate),^3^(intermediate ≠ high).

When compared to the lowest tertile of HDL-C, the highest tertile had more women, older ages, and, as expected, higher concentration of cholesterol. HL and CETP activities were reduced, and HL and PLTP increased in the upper tertile of HDL-C as compared to those in the lowest tertile. No differences were found in hsCRP and TNF-α. It is noteworthy that oxLDL-Ab levels were significantly higher in the high HDL-C group, in comparison to the low HDL-C group.

To explore the influence of the independent variables, HDL-C concentration, HDL-C tertiles, sex, ApoAI, ApoB, inflammatory markers and age, on the dependent variable, oxLDL-Ab, a linear regression analysis was performed, as shown in Table 2 . OxLDL-Ab levels were influenced by age, HDL-C, HDL-C tertiles, HDL-3C, and ApoAI.

**Table 2:** Simple linear regression using oxLDL-Ab as dependent variable

Independent variables	B (SE)	p values	R	R^2^
**Age**	0.002 (.001)	**0.027**	0.216	0.047
**Male**	8.66 (.029)	0.998	0.000	0.000
**HDL-C**	0.002 (.001)	**0.004**	0.293	0.086
**HDL tertiles**	0.042 (.015)	**0.006**	0.276	0.076
**HDL-2C**	0.004 (.002)	0.054	0.191	0.036
**HDL-3C**	0.002 (.001)	**0.016**	0.237	0.056
**ApoAI**	0.001 (.000)	**0.002**	0.308	0.095
**LDL**	0.000 (.000)	0.179	0.132	0.017
**ApoB**	0.001 (.000)	0.204	0.126	0.016
**hsCRP**	0.052 (.032)	0.105	0.177	0.031
**TNF-α**	0.002 (.001)	0.086	0.225	0.051
**CIMT**	0.146 (.085)	0.093	-0.25	0.316

Simple linear regression. ApoAI: apolipoprotein AI; ApoB: apolipoprotein B; C: cholesterol; CIMT: carotid intima-media thickness; HDL: high-density lipoprotein; hsCRP: high-sensitivity C-reactive protein; LDL: low-density lipoprotein; oxLDL-Ab: autoantibodies against oxidized low-density lipoprotein; TNF-α: tumor necrosis factor alpha.

In the adjusted regression analysis, only HDL-C and ApoAI were independently related to oxLDL-Ab levels in the model adjusted by the covariates of age and ApoB, as shown in Table 3 .

**Table 3:** Linear regression adjusted by covariates

Independent variables	Models	B (SE)	p values	R	R^2^
**HDL-C**	**HDL-C** Age	0.002 (.001)	0.044	0.30	0.090
	**HDL-C** ApoB	0.002 (.001)	0.011	0.279	0.078
**HDL-3C**	**HDL-3C** Age	0.001 (.001)	0.166	0.272	0.074
	**HDL-3C** ApoB	**0.002 (.001)**	0.054	**0.234**	**0.055**
**ApoAI**	**ApoAI** Age	0.153 (.059)	0.019	0.318	0.101
	**ApoAI** ApoB	0.001 (.00)	0.004	0.310	0.096

Adjusted linear regression. ApoAI: apolipoprotein AI; ApoB: apolipoprotein B; HDL-C: high-density lipoprotein cholesterol.

Linear regression curve models of HDL and ApoAI relationships to oxLDL-Ab are shown in Figures 1 and 2 , respectively.

**Figure 1:** Curve model: oxLDL-Ab versus HDL. HDL: high-density lipoprotein; oxLDL-Ab: autoantibodies against oxidized low-density lipoprotein.

**Figure 2:** Curve model: oxLDL-Ab versus ApoAI. ApoAI: apolipoprotein AI; oxLDL-Ab: autoantibodies against oxidized low-density lipoprotein.

## Discussion

Retention of oxLDL in the subendothelial layer of the arterial wall is an initiating step of atherosclerosis.^19^ OxLDL binds to scavenger receptors, such as Lox1 and SR-B1, to prompt downstream deleterious pathways that culminate in plaque progression.^20^ Beyond reverse cholesterol transport, HDL modulates humoral immunity of atherosclerotic plaque, upregulating IL5 and Th2 response, which are involved in B cell activation and release of oxLDL-Ab.^21^ Correspondingly, our study, for the first time, found an independent positive correlation between serum levels of HDL-C and oxLDL-Ab.

Prior experimental data have consistently demonstrated an atheroprotective role for oxLDL-Ab. From a mechanistic perspective, oxLDL-Ab colocalizes in the atherosclerotic plaque, where it binds to oxLDL epitopes forming immune complexes that cannot interact with macrophage Fcγ receptors.^13 , 22^ As a result, neutralization of oxLDL epitopes by oxLDL-Ab prevents macrophage activation, interrupting an imperative pathway of plaque progression.^13^ In line with this, Dai et al^22^ demonstrated that pretreatment of macrophages with oxLDL-Ab prevented oxLDL-induced cell death and NF-kappaB activation. Accordingly, treatment with oxLDL-Ab significantly reduced cross-sectional atherosclerotic plaque area and vascular cell adhesion molecule 1, and it mitigated macrophage uptake in LDLr^-^mice.^22 , 24^

Cumulative data from clinical studies have also supported a role for oxLDL-Ab as a marker of cardiovascular disease. In this matter, serum levels of oxLDL-Ab have consistently shown an independent inverse correlation with common carotid artery intima-media thickness and progression of carotid atherosclerosis.^16 , 25 - 30^ For example, in a cohort of 226 patients with hypertension prospectively enrolled in carotid ultrasound analysis, those with the lowest value of oxLDL-Ab showed a 3-fold reduced risk of any 4-year progression of intima-media thickness of the carotid arteries.^31^ Similarly, among individuals undergoing clinically indicated coronary angiography, those in the highest tertiles of oxLDL-Ab had a 37% lower risk of angiographically significant coronary atherosclerosis, and displayed a lower number of diseased arteries when compared to those with the lowest titers of antibodies.^32 , 33^ Consistently, Shoji et al.^34^ observed a 2-fold increased 5-year cardiovascular mortality among individuals with end-stage renal disease and low oxLDL-Ab, when compared to patients with end-stage renal disease with higher levels of oxLDL-Ab.

In addition to the aforesaid, we found a positive independent correlation between serum levels of HDL-C and oxLDL-Ab. From a mechanistic perspective, this finding may derive from immunomodulatory effects of HDL on Th2 response, which reasonably potentiates oxLDL-Ab release. This hypothesis still warrants further examination. Other potential reasons for the verified correlation may be highlighted, for example, experimentally, HDL attenuated uptake of oxLDL by macrophages. This may result in accumulation of oxLDL in the plaque microenvironment, favoring local humoral response.^35^

This study had some limitations. More importantly, we assumed that HDL induces oxLDL-Ab by modulating IL5-related Th2 response. Nevertheless, measurement of IL5 were not performed. Furthermore, oxLDL levels, which are closely related to oxLDL-Ab release, were also not assessed and would have been a reasonable adjustment variable in our models. Finally, sample size was relatively small, which may have jeopardized statistical power to claim correlation.

## Conclusion

Serum levels of oxLDL-Ab and HDL-C are positively related.
